# Does Eating Vegetables at Start of Meal Prevent Childhood Overweight in Japan? A-CHILD Study

**DOI:** 10.3389/fped.2018.00134

**Published:** 2018-05-17

**Authors:** Yukako Tani, Takeo Fujiwara, Manami Ochi, Aya Isumi, Tsuguhiko Kato

**Affiliations:** ^1^Department of Global Health Promotion, Tokyo Medical and Dental University (TMDU), Tokyo, Japan; ^2^Japan Society for the Promotion of Science, Tokyo, Japan; ^3^Japan Support Center for Suicide Countermeasures, National Institute of Mental Health, National Center of Neurology and Psychiatry, Tokyo, Japan; ^4^Department of Social Medicine, National Center for Child Health and Development, Tokyo, Japan

**Keywords:** eating behavior, order of food consumed, food sequencing, behavioral economics, childhood obesity

## Abstract

**Background:** Because eating behaviors are established early in life, it is important to instill healthy eating habits in children. However, no published studies have examined the effects of what is habitually consumed first at a meal on children's body weight in real settings. The aim of this study was to examine the associations between what was consumed (vegetables, rice/bread, meat/fish, or soup) at the start of a meal and childhood overweight in Japan.

**Methods:** We used cross-sectional data from the Adachi Child Health Impact of Living Difficulty (A-CHILD) study, a population-based study comprising all first-grade students in Adachi City, Tokyo, Japan, performed in 2015. Through a questionnaire, we identified what types of food children ate first at meals. The questionnaire was completed by 4,040 caregivers. We used corresponding school health check-up data (height and weight) to assess overweight in each child.

**Results:** The proportions of what was consumed first at a meal were 11.6, 23.3, 25.4, 9.8, and 29.9% for vegetables, meat/fish, rice/bread, soup, and undetermined (variable), respectively. Multivariate logistic regression showed the odds ratio of being overweight was 1.83 in children who ate meat/fish first (95% CI: 1.27–2.64, *p* < 0.01) compared with children who ate vegetables first. In contrast, the odds ratios in children who consumed rice/bread or soup first compared with children who ate vegetables first were 1.11 (95% CI: 0.76–1.61, *p* = 0.59) and 1.29 (95% CI: 0.83–2.01, *p* = 0.26), respectively.

**Conclusion:** Children who eat meat/fish at the start of a meal are more likely to be overweight than those who eat vegetables at the start of a meal. Future studies are needed to investigate the mechanisms of how the order in which food is consumed at a meal affects weight status in children.

## Introduction

The prevalence of childhood overweight and obesity has increased worldwide in recent decades and it has become a major public health epidemic (1–3). Between 1980 and 2013, the prevalence of overweight and obesity in developed countries has increased from 16.9 to 23.8% in boys and from 16.2 to 22.6% in girls (3). Overweight and obese children are likely to stay obese into adulthood (4–6), and have greater risk of heart disease, type 2 diabetes, stroke, and premature death and disability in adulthood (7, 8). Furthermore, children nowadays are more likely to become overweight at a younger age (9). Therefore, prevention of overweight in young children is important.

Although several risk factors for childhood overweight have been identified (10), the effectiveness of intervention programs has been minor and short-term (11). There is a need to establish a feasible and effective strategy against childhood overweight using an alternative approach. A notable example is the “CAN Approach,” which is based on the practice of making healthy foods more convenient, attractive, and normative. This method nudges healthy food choices through behavioral economic theories of decision-making (12). For example, placing the healthiest food first and the least healthy food last at a buffet spread nudged people to select healthier meals, due to a high percentage of people taking the first available food (13). Similarly, the order of food consumption at meals can be habitual in conjunction with mealtime socialization (14), and this seems to be a normative choice for children. Therefore, we hypothesized that eating vegetables first at meals may increase vegetable intake, which aids in the prevention of overweight.

The order of food consumption may have an effect on overweight by changing the amount of food consumed during the rest of the meal. Some controlled studies in adults have shown that eating low energy density food first (vegetables, soup, or fruit) reduces total meal energy intake (15–17). Furthermore, Roe et al. reported that consuming low energy density salad before, rather than with, the main course increases total vegetable consumption (18).

The order of food consumption may also affect overweight and obesity by changing postprandial digestibility and metabolic effects. Glycemic excursions and incremental glucose peaks are reportedly significantly smaller when vegetables are consumed before carbohydrate foods compared with the reverse sequence (19). This may be due to the fiber in vegetables, which affects the speed of digestion and the amount of insulin needed for subsequent metabolic disposal (20).

Eating patterns established in early life tend to persist into adolescence and adulthood (21–23). Therefore, it is important to instill healthy eating habits in children. To date, however, no published studies have examined the effects of order of food consumption at meals on children's body weight in real settings.

In Japan, a typical meal—known as “ichi-ju san-sai”—consists of a staple food (such as rice), a soup (usually miso), and three dishes (one main side dish and two side dishes). The food is served all at once (24). Therefore, the order of food consumption at a meal can vary significantly among individuals.

To that end, a health promotion strategy known as “Eat Vegetables First at Meals” was launched in Adachi City in Tokyo, Japan, in 2013 to address various health problems. Specifically, the municipality focused on children in public daycare centers. To assess the impact of this health promotion policy, a questionnaire survey concerning eating habits of children in Grade 1 in Adachi City was implemented. The responses to the questionnaire was linked with school health checks data, including objective assessments of height and weight.

The purpose of the present study was to use this population-based sample to examine the associations between the order of food consumption at a meal in Grade 1 elementary school children and their overweight status in Japan.

## Materials and methods

### Study design and subjects

We used data from the Adachi Child Health Impact of Living Difficulty (A-CHILD) study performed in Adachi City, Tokyo, Japan, in 2015 (25). The survey covered all 69 public elementary schools in Adachi City. Between July 2015 and November 2015, self-reported questionnaires with anonymous unique ID were distributed to 5,355 children in the first grade of elementary school (aged 6 to 7 years). Children were asked to pass on the questionnaires to their caregivers at home to fill out. The children then returned the completed questionnaires to their school. A total of 4,467 questionnaires were returned (response rate: 83.4%) and 4,291 of the responding caregivers provided informed consent to include their child in the study (25). Questionnaire responses were linked to each participating child's school health check-up data from the same year (linkage rate: 100%) to determine their body mass indexes (BMI). The analyses were carried out using the data of 4,040 participants after excluding the following: (i) potential participants whose sex, date of birth, or body weight status were missing (*n* = 189); and (ii) potential participants whose caregivers had not answered the questions about what was consumed first (*n* = 62). The A-CHILD protocol was approved by the Ethics Committee of the National Center for Child Health and Development (No. 1187). All participants gave written informed consent in accordance with the Declaration of Helsinki.

### Child body weight status

School teachers assessed elementary school children's height and weight during health check-ups according to a standardized protocol (26). Height was measured to the nearest 0.1 cm using a portable stadiometer, and weight to the nearest 0.1 kg on a digital scale without shoes and wearing light clothing. BMI was calculated from weight (in kilograms) over square of height (in meters). BMI was expressed as z-score representing the deviation in standard deviation units from the mean of a standard normal distribution of BMI specific to age and sex according to the standard of the WHO Child Growth Standards. Children were categorized as non-overweight or overweight using +1 standard deviation cut-off points (27).

### Order of food consumption

What was consumed first at meals over the past month was assessed using the following question: “What does your child eat first at mealtimes? Circle the answer that best applies for the past month.” The responses to this question were “vegetables,” “meat/fish,” “rice/bread (staple),” “soup,” or “not determined (variable).”

### Covariates

Because the “Eat Vegetables First at Meals” campaign was launched prior to our study, adjustments were made for the preschool status (public nursery, private nursery, or private kindergarten) of each child. Potential confounders of the association between what is consumed first at meals and overweight were assessed by questionnaires completed by caregivers.

The children's physical activity was assessed according to the frequency of being physically active for half an hour or more, and was categorized into four groups (never/rarely, 1–2, 3–4, or ≥5 times/week). The status of the responder to the questionnaire was categorized into two groups (mother or father).

Marital status of caregivers was categorized into two groups (married/common-law marriage or unmarried/divorced/bereavement). Annual normalized household income was determined from the household income and the number of household members and divided into three categories (<2.50, 2.50–3.49, or ≥3.50 million yen).

Family meal status during dinner (children eating with family or only with other children/alone) was included in the analysis because family meals are related to children's eating patterns and body weight status (28). Because children are largely cared for by their mothers and only 10% of fathers take care of children's meals in Japan (29), mother's characteristics were included: age (<30, 30–39, or ≥40 years), educational status (low: junior high school/high school; middle: technical/junior college/college dropout; or high: college/graduate school), and employment status (full-time, part-time, self-employed, side job, or unemployed). Participating children's BMI at age 3 years was calculated using the height and weight reported by their caregivers. BMI was expressed as z-score representing the deviation in standard deviation units from the mean of a standard normal distribution of BMI specific to age and sex according to the standard of the WHO Child Growth Standards. Children were categorized as non-overweight or overweight using +1 standard deviation cut points (30). Mothers' and fathers' BMI were calculated using their self-reported heights and weights. Standard categories of BMI (31) were used to characterize parents as obese (BMI ≥ 30.0 kg/m^2^), overweight (BMI = 25.0–29.9 kg/m^2^), normal (BMI = 18.5–24.9 kg/m^2^), and underweight (BMI < 18.5 kg/m^2^).

### Statistical analysis

First, χ^2^-tests were used to assess participants' characteristics and differences in what is consumed first at meals. Second, the association between what is consumed first at meals and the overweight status of each child was evaluated using logistic regression analysis to calculate the adjusted odds ratios (OR) with 95% confidence intervals (CI). The following sequence of models was constructed: Model 1 was adjusted for the sex of each child; Model 2 was additionally adjusted for each child's physical activity and preschool status, each respondent's (parent's) status and marital status, annual normalized household income, family meal, mother's age, mother's education and mother's employment; and Model 3 was further adjusted for each child's BMI at 3 years, and mother's and father's BMI. All analyses were conducted using STATA version 13 (Stata Statistical Software: Release 13. College Station, TX: StataCorp LP).

## Results

Overall, the proportions of what was consumed first at meals were 11.6, 23.3, 25.4, 9.8, and 29.9% for vegetables, meat/fish, rice/bread, soup, and not determined, respectively (Table 1). Of the participating children, 13.5% were overweight. Most respondents were mothers (90.7%). The proportions of preschool status were 14.0%, 25.9%, and 55.8% for private nursery, public nursery, and private kindergarten, respectively. Of the children who ate vegetables first at meals, 30.2% previously attended public nurseries, whereas 22.9% of children who ate meat/fish first at meals previously attended public nurseries.

**Table 1:** Characteristics of participating Japanese school children and their caregivers (*n* = 4040).

	**Total**		**Food consumed first**										**χ2**	***p*-value**
			**Vegetables**		**Meat/Fish**		**Rice/Bread**		**Soup**		**Not determined**			
	***n***	**%**	***n***	**%**	***n***	**%**	***n***	**%**	***n***	**%**	***n***	**%**		
**CHILD-RELATED CHARACTERISTICS**														
Food consumed first														
Vegetables	470	11.6	470	100										
Meat/Fish	943	23.3			943	100								
Rice/Bread	1,024	25.4					1024	100						
Soup	396	9.8							396	100				
Not determined	1,207	29.9									1207	100		
Sex														
Boy	2,089	51.7	213	45.3	571	60.6	540	52.7	192	48.5	573	47.5	48.0	<0.001
Girl	1,951	48.3	257	54.7	372	39.4	484	47.3	204	51.5	634	52.5		
Body weight status (BMI for age z score)														
Normal (<1SD)	3,495	86.5	419	89.1	772	81.9	895	87.4	342	86.4	1,067	88.4	24.6	<0.001
Overweight (≥1SD)	545	13.5	51	10.9	171	18.1	129	12.6	54	13.6	140	11.6		
Physical activity														
Never/rarely	410	10.1	34	7.2	91	9.7	116	11.3	44	11.1	125	10.4	18.8	0.28
1–2 times/weeks	1,832	45.3	216	46	408	43.3	494	48.2	177	44.7	537	44.5		
3–4 times/weeks	1,068	26.4	135	28.7	266	28.2	241	23.5	96	24.2	330	27.3		
≥5 times/weeks	718	17.8	83	17.7	175	18.6	169	16.5	78	19.7	213	17.6		
Missing	12	0.3	2	0.4	3	0.3	4	0.4	1	0.3	2	0.2		
Preschool status														
Public nursery	1,045	25.9	142	30.2	216	22.9	267	26.1	122	30.8	298	24.7	27.4	0.01
Private nursery	566	14	65	13.8	122	12.9	155	15.1	57	14.4	167	13.8		
Private kindergarten	2,253	55.8	241	51.3	560	59.4	558	54.5	193	48.7	701	58.1		
Other/none/missing	176	4.4	22	4.7	45	4.8	44	4.3	24	6.1	41	3.4		
Body weight status at 3 age years (BMI for age z score)														
Normal (<1SD)	2,804	69.4	327	69.6	656	69.6	707	69	269	67.9	845	70	4.8	0.78
Overweight (≥1SD)	691	17.1	80	17	171	18.1	168	16.4	65	16.4	207	17.1		
Missing	545	13.5	63	13.4	116	12.3	149	14.6	62	15.7	155	12.8		
**RESPONDENT'S STATUS**														
Mother	3,664	90.7	445	94.7	846	89.7	922	90	366	92.4	1,085	89.9	14.8	0.06
Father	308	7.6	20	4.3	84	8.9	82	8	24	6.1	98	8.1		
Other/missing	68	1.7	5	1.1	13	1.4	20	2	6	1.5	24	2		
**HOUSEHOLD-RELATED CHARACTERISTICS**														
Marital status of caregiver														
Married/common-law marriage	3,581	88.6	423	90	842	89.3	909	88.8	345	87.1	1,062	88	3.7	0.89
Unmarried/divorced/bereavement	338	8.4	35	7.4	76	8.1	86	8.4	35	8.8	106	8.8		
Other/missing	121	3	12	2.6	25	2.7	29	2.8	16	4	39	3.2		
Annual normalized household income (million yen)														
Low (<2.50)	1,557	38.5	164	34.9	352	37.3	388	37.9	150	37.9	503	41.7	17.2	0.14
Middle (2.50–3.49)	1,056	26.1	130	27.7	245	26	264	25.8	97	24.5	320	26.5		
High (≥3.50)	1,028	25.4	137	29.1	254	26.9	264	25.8	106	26.8	267	22.1		
Missing	399	9.9	39	8.3	92	9.8	108	10.5	43	10.9	117	9.7		
Family meal														
Children eat with family	3,890	96.3	454	96.6	903	95.8	981	95.8	386	97.5	1166	96.6	6.7	0.57
Children eat separately	145	3.6	15	3.2	40	4.2	41	4	9	2.3	40	3.3		
Missing	5	0.1	1	0.2	0	0	2	0.2	1	0.3	1	0.1		
**CAREGIVER-RELATED CHARACTERISTICS**														
Mother's age (years)														
<30	212	5.2	20	4.3	47	5	55	5.4	23	5.8	67	5.6	13.5	0.34
30–39	2,086	51.6	243	51.7	478	50.7	509	49.7	201	50.8	655	54.3		
≥40	1,639	40.6	193	41.1	402	42.6	429	41.9	158	39.9	457	37.9		
Missing	103	2.5	14	3	16	1.7	31	3	14	3.5	28	2.3		
Mother's education														
Junior high school/high school	1,447	35.8	145	30.9	334	35.4	364	35.5	149	37.6	455	37.7	16.3	0.18
Technical/Junior college/college dropout	1,663	41.2	191	40.6	402	42.6	424	41.4	167	42.2	479	39.7		
Collage/graduate school	819	20.3	118	25.1	181	19.2	203	19.8	71	17.9	246	20.4		
Other/missing	111	2.7	16	3.4	26	2.8	33	3.2	9	2.3	27	2.2		
Mother's employment														
Full-time	775	19.2	99	21.1	176	18.7	205	20	93	23.5	202	16.7	25.3	0.19
Part-time	1,498	37.1	167	35.5	351	37.2	385	37.6	159	40.2	436	36.1		
Self-employed	198	4.9	25	5.3	43	4.6	45	4.4	13	3.3	72	6		
Side work	77	1.9	5	1.1	18	1.9	23	2.2	6	1.5	25	2.1		
Not employed	1,388	34.4	163	34.7	332	35.2	337	32.9	117	29.5	439	36.4		
Other/missing	104	2.6	11	2.3	23	2.4	29	2.8	8	2	33	2.7		
Mother's BMI														
Underweight (BMI<18.5)	525	13	73	15.5	107	11.3	130	12.7	61	15.4	154	12.8	26.1	0.05
Normal (18.5≤BMI<25.0)	2,831	70.1	323	68.7	688	73	707	69	274	69.2	839	69.5		
Overweight (25.0≤BMI<30.0)	351	8.7	45	9.6	79	8.4	99	9.7	34	8.6	94	7.8		
Obesity(BMI≥30.0)	72	1.8	5	1.1	19	2	15	1.5	2	0.5	31	2.6		
Missing	261	6.5	24	5.1	50	5.3	73	7.1	25	6.3	89	7.4		
Father's BMI														
Underweight (BMI<18.5)	83	2.1	12	2.6	19	2	24	2.3	6	1.5	22	1.8	14.8	0.54
Normal (18.5≤BMI<25.0)	2,330	57.7	282	60	542	57.5	590	57.6	234	59.1	682	56.5		
Overweight (25.0≤BMI<30.0)	856	21.2	96	20.4	210	22.3	217	21.2	80	20.2	253	21		
Obesity (BMI≥30.0)	144	3.6	7	1.5	36	3.8	31	3	18	4.5	52	4.3		
Missing	627	15.5	73	15.5	136	14.4	162	15.8	58	14.6	198	16.4		

*BMI, body mass index; SD, standard deviation*.

After adjusting for each child's sex, children who ate meat/fish first at meals were 1.82 times (95% CI: 1.30–2.54, *p* < 0.001) more likely to be overweight than those who ate vegetables first at meals (Model 1, Table 2). In contrast, the OR in children who ate rice/bread or soup first at first compared with children who ate vegetables first at meals were 1.18 (95% CI: 0.84–1.67, *p* = 0.34) and 1.30 (95% CI: 0.86-1.95, *p* = 0.21), respectively (Model 1, Table 2). Adjusting for each child's physical activity, preschool status, each respondent's (parent's) status and marital status, annual normalized household income, mother's age, mother's education, mother's employment and family meal, the OR in children who ate meat/fish first was slightly lower, but remained significant (1.80; 95% CI: 1.28–2.54, *p* < 0.01) (Model 2). Subsequent adjustment for each child's BMI at age 3 years and mother's and father's BMI did not weaken the association (1.83; 95% CI: 1.27–2.64, *p* < 0.01) (Model 3).

**Table 2:** Adjusted odds ratios for overweight status according to the food consumed first by Japanese school children (*n* = 4040).

	**Model 1**		**Model 2**		**Model 3**	
	**Overweight**		**Overweight**		**Overweight**	
	**OR (95%CI)**	***p*-value**	**OR (95%CI)**	***p*-value**	**OR (95%CI)**	***p*-value**
**FOOD CONSUMED FIRST**						
Vegetables	Reference		Reference		Reference	
Meat/Fish	1.82 (1.30–2.54)	<0.001	1.80 (1.28–2.54)	<0.01	1.83 (1.27–2.64)	<0.01
Rice/Bread	1.18 (0.84–1.67)	0.34	1.14 (0.80–1.61)	0.46	1.11 (0.76–1.61)	0.59
Soup	1.30 (0.86–1.95)	0.21	1.23 (0.81–1.85)	0.33	1.29 (0.83–2.01)	0.26
Not determined	1.08 (0.77–1.51)	0.67	1.06 (0.75–1.50)	0.74	0.99 (0.68–1.43)	0.95

*OR, odds ratio; CI, confidence interval. Model 1: Adjusted for child's sex. Model 2: Model 1 + adjusted for child's physical activity, preschool status, respondent status, marital status, annual normalized household income, family meal, mother's age, mother's education and mother's employment. Model 3: Model 2 + adjusted for child's body weight status at age 3 years, mother's and father's BMI*.

## Discussion

In this study, we found that children who habitually eat meat/fish first at meals are more likely to be overweight than those who eat vegetables first. In contrast, the effects of eating rice/bread or soup first on overweight did not differ significantly from that of eating vegetables first. To our knowledge, this is the first study to examine the effect of what is consumed first at meals on body weight status in children in a real setting.

There are several possible explanations for our findings. One possibility is that eating low energy density food (vegetables) first at meals may better promote satiety and reduce energy intake over the course of the meal compared with eating high energy density food (meat/fish) first. Indeed, a suggested strategy for enhancing satiety and reducing energy intake is to consume a filling portion of low energy density food, such as vegetables or salad, as a first course (32). Furthermore, in a single-meal study, serving children a low energy density, vegetable-based soup as a first course was associated with less energy intake from the more energy dense main course (33). In adults, consuming low energy density salads is associated with less energy intake over a meal than having no first course (15).

These findings are consistent with our present study, which found that the effect on overweight of eating soup, a low energy-density food, first did not differ significantly from that of eating vegetables first. In addition to vegetables, eating other low energy density foods (such as soup or fruit) as a first course is also associated with eating less of a higher energy density main course and less energy intake over the course of the meal (16, 17). In several multiple-meal studies of both adults and children, reducing the energy density of multiple meals over 2 days resulted in decreased cumulative energy intake (34, 35), suggesting that reducing the energy density of multiple meals leads to persistent and habitual decreases in energy intake from meals. This may in turn contribute to the prevention of overweight. Future studies are needed to investigate energy intake over a meal to clarify the mechanisms of the effect of food sequencing on overweight.

Another possibility is that eating vegetables first provides greater opportunity to eat more vegetables. In a crossover design, serving vegetables or vegetable-based-soup as a first course was associated with increased total vegetable consumption over the meal by children (33, 36). Children typically like fatty and sugary foods and dislike vegetables (37) because they innately prefer sweet and salty foods and reject sour and bitter foods (38). One Japanese study showed that vegetables were the most disliked of all foods in school lunches and that dislike was the main reason for leftovers among primary school children (39). Therefore, children who eat meat/fish first may leave more vegetables than those who eat vegetables first.

Furthermore, our measure of what was consumed first at meals may act as a proxy for the habitual quality of family meals. For example, the frequency of vegetables being served as part of family meals may be higher among children who eat vegetables first, and may in fact result in these children acquiring the habit of eating vegetables first. We found the prevalence of eating vegetables at both breakfast and dinner (over a month) was higher in children who ate vegetables first than in those who ate meat/fish first (48 vs. 31%, respectively; *p* < 0.001). Additionally, the consumption of obesity-related foods (juice, dessert, etc.) may be higher in children who eat meat/fish first. Further studies on the habitual quality of meals are needed to explore this possibility.

Eating rice/bread first at meals did not differ significantly from eating vegetables first in terms of the impact on overweight. Because eating vegetables before carbohydrates is associated with smaller glycemic excursions and glucose peaks than the reverse sequence (19), we hypothesized that eating vegetables first at meals would better protect against overweight than eating carbohydrates (rice/bread) first. However, the effects of eating vegetables first may not have depended on digestibility and metabolic effects in the present setting. The mechanisms underlying the differences in effects of what is consumed first still require further investigation.

This study has several limitations. First, we did not explore the validity or reliability of the question assessing what is consumed first at meals. We collected information on what children ate first from their caregivers. Therefore, the responses may have been biased due to the caregivers' perception. Social desirability bias may also have skewed the caregivers' responses. Nonetheless, we confirmed what was consumed first did not differ according to the caregiver's social status (marital status, income, mother's age, education, and employment), and the prevalence of eating vegetables first was higher in children from public nurseries, where a habituation policy of eating vegetables first had been implemented. Second, we lacked information on the amount of food that was consumed first. Therefore, we included children who ate both large and small amounts of vegetables first in the same category. For example, the “vegetables first” category may include children who had a bite of vegetables at the beginning of meals but ate the rest of the vegetables later. However, our hypothesis is not about the effectiveness of the *amount* of vegetables consumed first, but rather the *habit* of eating vegetables first, which leads to a higher frequency of eating vegetables. Future studies should aim to elucidate the mechanism by which habitually eating vegetables first increases the frequency or amount of vegetables consumed, which eventually prevents overweight among children. Third, because we lacked information on factors such as children's food preferences and caregivers' knowledge about nutrition, we may not have taken all confounding factors into account. For example, caregivers who know a lot about nutrition may not only pay attention to what their children eat, but may also teach them to eat vegetables first. Fourth, despite the difference in the nutritional value of fish and meat (chicken, red meat, etc.), we grouped these food categories together. Future study should divide these categories to clarify the mechanism of the effect of eating meat and fish first on overweight. Finally, we did not assess energy intake or physical exercise, and the study design was cross-sectional. Hence, the mechanism by which the habit of eating vegetables first prevents overweight among children is yet to be uncovered.

In conclusion, what was consumed first at meals was associated with children's body weight status. Based on the theories of behavioral economics, the habitual behavior of avoiding meat/fish first may nudge children to eat better. This strategy may be useful for “non-course-meal” culture in which all dishes are served at one time, such as the meals typically served in Japan. Further studies measuring total daily and mealtime energy intake, energy per food category, energy expenditure, food preferences, glucose metabolism, and satiety responsiveness based on food group order are warranted to determine whether and how eating vegetables first actually prevents childhood overweight.

## Author contributions

YT and TF conceived the design. TF, MO, AI, and TK collected data. YT reviewed literature and analyzed data. YT wrote first draft of paper. TF revised the first draft. MO, AI, and TK edited the manuscript. All authors approved the final version of the manuscript.

### Conflict of interest statement

The authors declare that the research was conducted in the absence of any commercial or financial relationships that could be construed as a potential conflict of interest.
